# Resilience concepts in psychiatry demonstrated with bipolar disorder

**DOI:** 10.1186/s40345-017-0112-6

**Published:** 2018-02-09

**Authors:** David G. Angeler, Craig R. Allen, Maj-Liz Persson

**Affiliations:** 1 Department of Aquatic Sciences and Assessment, Swedish University of Agricultural Sciences, PO Box 7050, 750 07 Uppsala, Sweden; 2 U.S. Geological Survey, Nebraska Cooperative Fish and Wildlife Research Unit, School of Natural Resources, University of Nebraska–Lincoln, Lincoln, NE USA; 3 PRIMA Adult Psychiatric Ward, Katrinebergsvägen 6, 117 43 Stockholm, Sweden

**Keywords:** Ecological resilience, Engineering resilience, Stress-recovery, Bipolar disorder, Mental disorders, Ecological theory, Interdisciplinary research

## Abstract

**Background:**

The term resilience describes stress–response patterns of subjects across scientific disciplines. In ecology, advances have been made to clearly distinguish resilience definitions based on underlying mechanistic assumptions. Engineering resilience (rebound) is used for describing the ability of subjects to recover from adverse conditions (disturbances), and is the rate of recovery. In contrast, the ecological resilience definition considers a systemic change: when complex systems (including humans) respond to disturbances by reorganizing into a new regime (stable state) where structural and functional aspects have fundamentally changed relative to the prior regime. In this context, resilience is an emergent property of complex systems. We argue that both resilience definitions and uses are appropriate in psychology and psychiatry, but although the differences are subtle, the implications and uses are profoundly different.

**Methods:**

We borrow from the field of ecology to discuss resilience concepts in the mental health sciences.

**Results:**

In psychology and psychiatry, the prevailing view of resilience is adaptation to, coping with, and recovery (engineering resilience) from adverse social and environmental conditions. Ecological resilience may be useful for describing vulnerability, onset, and the irreversibility patterns of mental disorders. We discuss this in the context of bipolar disorder.

**Conclusion:**

Rebound, adaptation, and coping are processes that are subsumed within the broader systemic organization of humans, from which ecological resilience emanates. Discerning resilience concepts in psychology and psychiatry has potential for a mechanistically appropriate contextualization of mental disorders at large. This might contribute to a refinement of theory and contextualize clinical practice within the broader systemic functioning of mental illnesses.

## Background

The concept of resilience has increased in use across scientific disciplines (e.g., ecology, social sciences, health sciences, etc). However, with the increased use of the concept, different definitions have been forwarded (Angeler and Allen 2016). These definitions often have different meanings but are frequently used interchangeably within and across scientific disciplines. This results, in many cases, in the loss of clarity of resilience concepts (Brand and Jax 2007), and may lead to an improper characterization of pattern–process (stress–response) relationships.

In psychology and psychiatry, resilience is very broadly defined as an individual’s positive *adaptation* (for definition of terms in italics see Table 1) to life tasks under stressful and adverse social situations (Goldberg and Williams 1988; Luthar 2003; Haddadi and Besharat 2010; Pęciłło 2016). Resilience is positive growth or adaptation that mediates rates of *recovery* following periods of homeostatic disruption (Richardson 2002). The concept describes a dynamic process of a person’s *coping capacity* related to risk factors. Risk factors are stressful life events (e.g., health problems, financial hardship, or problems at work or with family relationships; Rutter 2008) and an increased possibility of a person to develop a mental health condition (e.g., inherited vulnerability from a parent; Duffy et al. 2016).

**Table 1:** Overview and definitions of terms used in this paper adapted from Angeler and Allen (2016)

Term	Definition
Adaptation (Psychology)	Psychological adaptation is the dynamic process, grounded in a person’s intellect and emotions, which maintains a balance in their mental and emotional states, and in their interactions with their social and cultural environments
Recovery (Engineering resilience)	Engineering resilience is synonymous to recovery and focuses on the return of structural and functional attributes of systems to pre-disturbance conditions following a disturbance. The unit of measurement is time of recovery. This definition assumes that systems are characterized by a single equilibrium and therefore fails to account for the potential for alternative regimes of the same system. In bipolar disorder, recovery from a depression or (hypo)manic episode can be regarded as engineering resilience
Coping capacity	The ability of patients to use available resources (clinical practices), skills (learning) and awareness (self-knowledge) to face and manage adverse situations. The strengthening of coping capacities is a means to build resilience to the effects of mental health symptoms and stressful social and other external situations
Response ability	Response ability is a combination of awareness and capability, which is influenced by people’s personalities. Capabilities can be any form of intellect or a physical aspect, and awareness any form of knowledge and experience. Increasing awareness and capability result in increased response ability and determine the speed and magnitude of recovery from, and adaptation to, stressful situations
Ball-in-cup heuristic	This model is commonly applied in ecology to demonstrate resilience concepts. The possibility of complex systems to exist in alternative regimes is shown by different cups. The shape of the cups symbolizes the basin of attraction (stability characteristics of these alternative regimes): deeper and wider cups symbolize a higher resilience of an alternative regime relative to cups that are smaller and shallower. The cup shape can be considered analogous to people’s personalities that influence their adaptation and coping abilities with, for instance, bipolar disorder (see text). The ball symbolizes dynamic stress–response patterns: (1) engineering resilience after disturbances (i.e., when the ball stays within the cup, and (2) ecological resilience when a disturbance threshold is passed (i.e., when the ball rolls to another cup))
Alternative regime	A potential alternative configuration in terms of structural and functional patterns and processes of a system. Alternative regimes are explicit in ecological resilience. In bipolar disorder, the healthy and diseased states can be considered alternative regimes
Ecological resilience	Ecological resilience is a measure of the amount of stress needed to change a complex system from one set of processes and structures to a different set of processes and structures. In bipolar disorder, it is the amount of stress needed to change a patient’s health status from a healthy regime to a permanently diseased regime
Regime shift	A shift in regime is a persistent change in the structure, function, and mutually reinforced processes or feedbacks of a complex system. The change of regimes, or the shift, usually occurs when a change in an internal process (feedback) or a disturbance (external shock) triggers a completely different system behavior. In bipolar disorder, a regime shift occurs when the disorder is triggered in a person
Coerced resilience	Management interventions in an undesired regime to approach conditions of a desired regime. In bipolar disorder, clinical treatment ameliorates symptomatology and aims at approximating conditions of healthy individuals. Coerced resilience means that (1) permanent treatment is needed, (2) that treatment does not restore a healthy regime, and (3) that cessation of treatment restores the full-blown symptomatology of bipolar disorder
Stable equilibrium	Relatively stable system dynamics, which are controlled by a specific set of structural and functional patterns, processes, and feedbacks. In bipolar disorder, mood swings comprise stable equilibrium dynamics within the diseased regime
Emergent properties	A complex systemic feature that cannot be explained by the sum of individual system components. In bipolar disorder, the diseased regime emerges from the complex interplay between genetic, physiological, brain-structural, behavioral, and personality traits of patients, and their interactions with social and environmental factors. This complex interplay determines the symptomatology and their recurrence dynamics
Feedbacks	In ecological systems feedbacks arise from the set of interactions between patterns and processes. Feedbacks control an effect by influencing and being influenced by the process which gave rise to it. A positive feedback enhances or amplifies these processes, while negative feedbacks have the opposite effects. Positive and negative feedbacks generally do not imply any judgment of value regarding the desirability of the effects or outcomes (e.g., healthy vs. diseased regime in bipolar disorder). Positive feedbacks are of most interest in resilience theory; these help maintain structure, function, and processes in specific alternative regimes

Resilience is often conceptualized as existing along a continuum of vulnerability. Individuals with low vulnerability have a high resistance to psychopathology, although they are not entirely invulnerable to the development of a psychiatric disorder (Goldberg 1972). Such resistant individuals are often referred to as being resilient (e.g., Tiet et al. 1998). This resilience can be related to the concept of *response ability*, which has been defined as a combination of awareness and capability (Campbell 2011). Increasing awareness and capability result in increased response ability. It is determined by people’s personalities, which influence their capability (i.e., any form of intellect or a physical aspect) and awareness, which depends on their knowledge, learning and experience (Campbell 2011). In addition to individual´s personality traits (neuroticism, hyperthymia, extravertedness, humor) other factors (social support, harsh childhood) influence resilience. The complex combination of factors determines whether or not, or how fast people recover from stressful life events (Bonanno 2004; Bonanno and Mancini 2008). That is, although some people resist stressful events, others need time to recover competent functioning, while others may permanently suffer from such events (for instance, when incurable bipolar disorder is triggered).

**Fig. 1:**
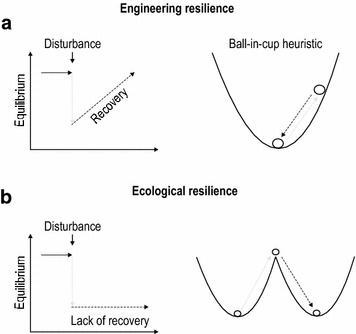
Schematic distinguishing between (**a**) recovery (engineering resilience) and (**b**) ecological resilience. Panels on the left show subject (humans, ecosystems) trajectories before (full black arrow), during (gray arrow), and after disturbances (black broken arrow). Panels on the right express these dynamics with ball-in-cup heuristics commonly used in ecology. In the case of recovery/engineering resilience, the ball rolls back to its equilibrium position after a disturbance. In the case of ecological resilience, the ball rolls over the cup’s brink and falls into a new cup. This cup represents an alternative stable system regime from which recovery to the previous regime is impossible. This is symbolized with the ball not rolling back to the previous cup

A concept related to adaptation, coping, and response ability in the mental health sciences is “*engineering resilience*” in ecology. Engineering resilience is synonymous with recovery, bounce back and resiliency (Angeler and Allen 2016), and is frequently shown as a *ball*-*in*-*cup heuristic* (Fig. 1a). In this heuristic, recovery is symbolized with the ball moving away from and then returning to its original position after disturbances (Fig. 1a). Recovery, or engineering resilience, is a dynamic process of both persons and ecosystems in response to disturbances, and the speed and the magnitude of recovery from stressful events depend on traits in both cases (response ability in people [Campbell 2011]; biodiversity in ecosystems [Nash et al. 2016]).

It follows that inherent in the psychological and psychiatric view of resilience is one’s ability to adapt to, cope with and bounce back from a negative experience by restoring well-being and social and self-functioning of individuals. That is, in psychology and psychiatry, coping, adaptation, and recovery are linked resilience aspects used to describe patients’ abilities to deal with adverse conditions. However, sometimes subjects suffer chronically from traumatic events. In such cases, implications of resilience concepts and treatment alternatives are quite different. When a full recovery of subjects is not tenable, treatment is reactive and focuses on alleviating symptoms of mental illness through medication and therapy (developing adaptation and coping skills). This suggests that the engineering resilience, adaptation, and coping aspects of resilience are useful for describing the ability of patients to deal with symptoms of mental disorders. However, these concepts frequently fail to characterize the broader systemic aspects of stress–response patterns related to psychological and psychiatric phenomena, an observation true in ecology as well.

Ecologists have become increasingly aware that ecological (e.g., lakes) and other complex systems of people and nature frequently do not bounce back after disturbances (Allen et al. 2014). Instead, when their adaptive capacity to stress is exhausted, they reorganize in an *alternative system regime* once a disturbance threshold has been passed (Fig. 1b). An alternative system regime means that basic structural (e.g., species assemblages) and functional patterns and processes (e.g., production of food and fiber) are distinctly different compared to the system regime that existed prior to passing the disturbance threshold. Ecologist use the term “*ecological resilience*,” when a system reorganizes in an alternative regime rather than bounces back after disturbances (Holling 1973).

There are striking similarities between ecological systems and humans in terms of the complexity of responses to adverse conditions (disturbances). In this paper, we point out the utility of the ecological resilience definition for describing some psychological and psychiatric phenomena; for instance, bipolar disorder. We contend that similar to ecological systems, resilience in bipolar disorder and other mental illnesses is an emergent phenomenon, which allows for the existence of alternative healthy and diseased regimes in patients that develop disorders. That is, both the systemic complexity of bipolar disorder (vulnerability, onset, irreversibility) and clinical interventions to ameliorate symptoms of the disorder well align with the ecological resilience definition. We argue that engineering resilience, adaptation, and coping are processes that are subsumed within the broader systemic organization of bipolar disorder that ecological resilience characterizes, and that these different definitions of resilience are therefore not mutually exclusive. We contend that discerning ecological and engineering resilience, and related concepts (adaptation, coping) in psychology and psychiatry, has potential for a mechanistically appropriate contextualization of mental disorders and psychological phenomena at large. This might contribute to a refinement of theory in the mental health sciences and contextualize and unify clinical practice (preventative *vs* reactive treatment) within the broader systemic functioning of mental illnesses.

## Bipolar disorder and resilience

Bipolar or manic–depressive disorder is an affective disorder characterized by pronounced mood swings with recurrent cycles of (hypo)mania (increased energy levels, decreased need for sleep, racing thoughts, pressure of speech, frequent agitation, confusion and distraction, heightened libido, and in extreme forms, hallucinations and delusions), and severe depression episodes (chaos, emotional emptiness, despair, self-stigma, doom, anhedonia, guilt, monochromatic world view, suicidal ideology) (Goodwin and Jamison 2007). The disorder comprises a spectrum wherein (hypo)manic and depression symptoms manifest with high variability and magnitude among patients, and these symptoms often co-occur (mixed states) (Phelps 2006). The illness affects between 3 and 8% of the human population (Goodwin and Jamison 2007), although these numbers may be higher because current diagnostic problems complicate differentiating between unipolar and bipolar depression (Bauer and Pfennig 2005).

**Fig. 2:**
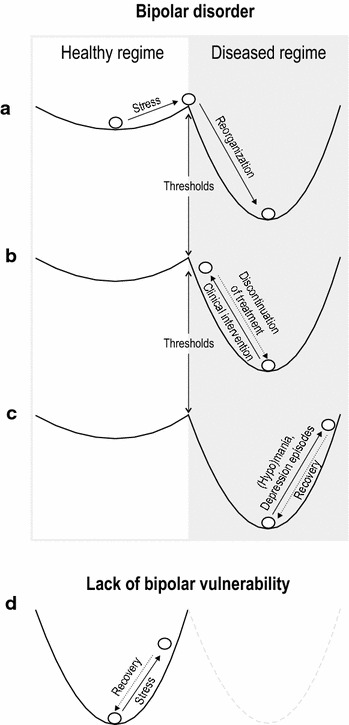
Ball-in-cup heuristic in the context of bipolar disorder. **a** Individual with bipolar vulnerability (symbolized with the shallow cup). Stressful life events trigger the chronic, incurable disorder (symbolized with the ball rolling from the healthy into a new (diseased) regime once a stress threshold has been passed). **b** Bipolar individual receiving healthcare (medication, therapy). Clinical intervention does not restore the regime prior to the outbreak of the disorder (no recovery). Instead, it ameliorates symptoms and increases personal and interpersonal functioning of the patient within the diseased regime (full arrow). Discontinuing clinical treatment (dotted arrow) eventually restores the full symptomatology. **c** Engineering resilience subsumed within ecological resilience: shown are stress-recovery patterns related to episodes of (hypo)mania and depression in the diseased regime. **d** Individual without bipolar vulnerability (i.e., in a healthy regime) recovering from adverse situations (stress). The lack of vulnerability is also symbolized by the absence of a diseased regime (gray dotted cup). Note that similar to persons without disposition to bipolar disorder, stress–response dynamics also occur in the healthy regime of patients with bipolar disorder (not shown in the figure)

Bipolar disorder, like many other mental disorders, has an underlying genetic component that increases the vulnerability to, and triggers, the disorder, frequently in adolescence or early adulthood, and often in response to stressful life experiences (Goodwin and Jamison 2007). This vulnerability can be visualized with a shallower bottom in the ball-in-cup heuristic (Fig. 2a-c), relative to a person without this vulnerability (deeper bottom of cup in Fig. 2d). That is, the heuristic exemplifies that people without bipolar vulnerability are more likely to rebound from stressful life events (engineering resilience) compared to people vulnerable to bipolarity. Note that, for simplicity, Fig. 2 is meant for demonstration only and therefore does not represent bipolar disorder as a spectrum illness with different degrees of people’s vulnerability to develop the disorder (Phelps 2006).

Given the susceptibility of people with disposition to bipolar disorder, the illness becomes eventually expressed after a threshold of stressful live events has been passed. This can be symbolized with the ball rolling into another cup (Fig. 2a). This indicates that the patient has undergone a “*regime shift*,” whereby his or her functioning becomes critically conditioned by the symptomatology of the disorder after this shift. Because bipolar disorder has no cure, the ball-in-cup heuristic is useful for highlighting that the patient has moved to a permanent (diseased) regime from which recovery to the symptom free health status prior to the outbreak of the disorder is highly unlikely (Fig. 2b).

Medication and different forms of therapy (psychotherapy, cognitive behavioral therapy, exercise) are common approaches to treat the symptoms of bipolar disorder (Phelps 2006). Although effective in terms of improving patients personal and interpersonal functioning, they do not cure the illness. That is, there is no full “functional restoration” of patients that would indicate recovery from the disorder, despite clinical interventions (i.e., return from the deep to the shallow cup in Fig. 2b). Psychiatrists are well aware that clinical treatment ameliorates the symptoms of bipolar disorder, rather than fully restores cognitive, behavioral, and functional characteristics prior to those before the onset of the disorder. From an ecological resilience point of view, clinical interventions can be considered a “coercion” of the diseased regime [*coerced resilience* (Rist et al. 2014)], by targeting the approximation of desired functionality of patients comparable to healthy individuals. This coercion is symbolized by the full arrow in Fig. 2b pushing the ball close to the regime before the outbreak of the disorder. That treatment is only a coerced condition of the disease regime is evident by the well-known fact that, for instance, breakthrough depression events can occur despite clinical treatment (Miklowitz and Gitlin 2015). These breakthrough depressions are indicative of the stability of the diseased regime. The notion of clinical treatment of bipolar disorder being a coerced condition of the diseased regime also helps explain why individuals that cease medication revert to express the full-blown symptomatology of (hypo)manic and depressive episodes of the disorder. This is symbolized with the dotted arrow in Fig. 2b. This further indicates the stability of the diseased regime.

Such coerced regimes are also observed in ecological systems. In boreal lakes, acidified rain has altered the pH, a measure of acid content, of lakes necessitating expensive and difficult additions of lime to restore pH levels toward neutrality (Angeler and Goedkoop 2010). However, like bipolar human subjects, the rehabilitation is temporary and requires constant management input (lime treatment). Cessation of liming often leads to the reestablishment of the acidified lake conditions (Clair and Hindar 2005).

Following the ecological resilience definition, the acidified regimes of boreal lakes and the diseased regime of bipolar disorder are systemic features wherein patterns and processes operate in a *stable equilibrium*. In bipolar disorder, these pattern–process relationships are manifested in the expression of symptoms (patterns) and their recurrence (dynamic process). The deviations from relative symptom free periods to episodes of depression or (hypo)mania that are inherent in the mood swings, whether or not the patient receives clinical treatment, are a central part in this dynamic process. These deviations, which impair the patients functioning, are followed by periods of recovery whereby the patient’s health improves. This recovery is indicative of engineering resilience, and because recovery patterns differ between patients as a function of their coping and adaptation potential, recovery time (i.e., recovery as a process rate) can vary substantially. Taken together, this suggests that engineering resilience is subsumed within ecological resilience, meaning that deviations and recovery from equilibrium conditions can occur within both the healthy and diseased alternative regimes (Fig. 2c, d). However, engineering resilience fails to describe the return from the diseased regime to the healthy regime (Fig. 2a).

**Fig. 3:**
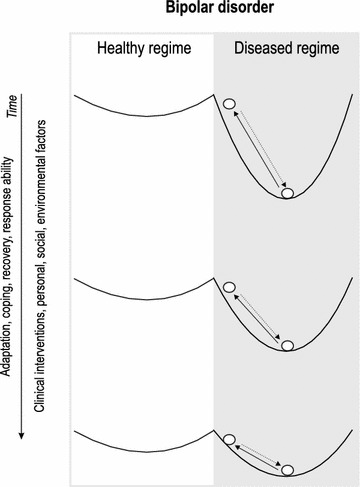
Ball-in-cup heuristic showing how clinical practices among other factors can contribute to improve patients’ capacities to cope with, adapt to, and recover from mood episodes in the coerced state in the diseased regime of bipolar disorder by modulating the shape of the cup (basin of attraction)

Understanding engineering resilience as a rate of recovery is mechanistically relatively simple and easy to measure. In contrast, ecological resilience as an *emergent systemic property* is a highly complex phenomenon, which complicates the prediction when a shift to an alternative regime can occur. This complexity results from the interaction of many factors that create positive *feedbacks* that are critical in maintaining ecological resilience (Angeler and Allen 2016). The cessation of treatment leading to a return of symptoms in the bipolar disorder or the increase in acidity in the boreal lake examples suggests that in both cases, we are able to manage engineering resilience (with clinical interventions or liming, respectively), but that we still lack the ability and knowledge to fully restore the feedbacks that would lead to a self-maintaining healthy regime in bipolar patients or a non-acidified, circumneutral regime of lakes. However, in bipolar disorder, different forms of psychotherapy, including psychoeducation, cognitive behavioral therapy and mindfulness-based cognitive therapy, have positive effects in terms of increased time to mood episode relapse or recurrence and improved depressive and anxiety symptoms (Salcedo et al. 2016). Similarly, adjunctive psychotherapy can be effective in treating manic symptoms (Miklowitz 2008), which confers significant advantages in preventing hospitalization for a mood episode (Solomon et al. 2008). Studies also highlighted the relevance of age, reporting faster functional recovery of older patients from a manic episode after first hospitalization relative to younger individuals (Tohen et al. 2000, 2003). Other factors such as shorter duration of the illness, higher social class, and treatment compliance may also influence recovery (Keck et al. 1998). Taken together, these observations suggest that clinical approaches (therapy, medication, exercise, mindfulness) together with environmental and personal factors may potentially gradually change some of the emergent properties of bipolar disorder (e.g., self-awareness of the precursor of the disorder, skills to prevent relapses). In turn, this might modulate the feedbacks of the diseased regime and influence resilience to bipolar episodes. This change of feedbacks and emergent properties (adaptation, coping ability) can be symbolized with a changing cup shape in the ball-in-cup heuristic (Fig. 3). The decreasing depth of the cups over time symbolizes that coping capacities and adaptation are strengthened and engineering resilience increased. This is shown with shorter arrows to maintain the coerced state of relative well-being in the diseased regime of bipolar disorder (Fig. 3).

## Conclusion

This paper argues that engineering and ecological resilience definitions used in ecology are appropriate in psychology and psychiatry, and although the differences are subtle, their implications and uses are profoundly different. Resilience concepts used in the mental health sciences that focus on dynamic processes (engineering resilience or rebound, adaptation to, and coping with stressful events) are subsumed within ecological resilience, an emergent phenomenon that emphasizes the existence of alternative regimes in bipolar disorder. In this context, deviations from and recovery to equilibrium conditions (engineering resilience) can be observed within the healthy and diseased regimes of patients. However, engineering resilience, adaptation, and coping capacity fail to capture the nonlinear dynamics that occur when a patient enters an alternative mental health regime, and the permanence or semipermanence of alternative regimes. This misrepresents the dynamics of complex systems, such as ecosystems and human brains.

Bipolar disorder can be considered the emergent phenomenon of the existence and interactions of a healthy and diseased regime. From a normative perspective, the healthy regime is desired, and the diseased regime undesired. In this context, the ecological resilience concept provides advantages over other resilience definitions to contextualize theory and management of the disorder, which differs between the healthy and the diseased regimes. Management of the healthy regime mainly requires proactive, preventative approaches to recognize bipolar vulnerability, reduce the risk of the disorder to become triggered, and foster the resilience of the healthy regime. Brain-structural and functional assessments are available to assess such risks (Frangou 2012; Frangou et al. 2017; Singh et al. 2014). For instance, Frangou (2012) showed that increased brain insular volume, decreased activation within the posterior and inferior parietal regions, and reduced fronto-insular and fronto-cingulate connectivity were risk factors in relatives of bipolar patients. Similarly, atypical patterns of prefrontal and subcortical intrinsic connectivity have been found in the healthy offspring of parents with bipolar disorder (Singh et al. 2014). There is also accruing evidence of a number of measurable and potentially modifiable markers of vulnerability and developing illness in youth at familial risk for bipolar disorder. These markers include in some high-risk children of bipolar-affected parents (Duffy et al. 2016): (1) sleep and anxiety disorders that precede mood disorders by several years and reflect an increased vulnerability, (2) early exposure to adverse situations like exposure to illness or neglect from a parent, (3) an increased risk of psychopathology manifested at behavioral and biological levels resulting from increased stress reactivity, (4) interrelated risk factors stemming from psychological processes (reward sensitivity, unstable self-esteem, rumination, and positive self-appraisal are risk factors for mood disorders), and (5) risk related to disturbances in circadian rhythm and immune dysfunction. Identifying such markers can be used for the development of specific early interventions that might reduce the risk of bipolar disorder to become triggered in people, particularly in youths with a high vulnerability (Duffy et al. 2016). Early warnings of bipolar vulnerability are especially required for youth because early onset of bipolar disorder might herald a more severe disease course in terms of chronicity and comorbidity (Perlis et al. 2004). In contrast, the diseased regime requires reactive approaches to ameliorate symptoms of the disorder and improve personal and interpersonal functioning of patients through different forms of therapy, medication, and exercise. These approaches have proven very useful to improve patients’ coping capacities and adaptation abilities, and facilitate faster recovery from episodes.

In summary, ecological resilience characterizes the broader systemic complexity and functioning of bipolar disorder and allows contextualizing resilience definitions. The ecological resilience definition emphasizes that both adaptation within single regimes and transformation between regimes, rather than adaptation alone, are critical elements in the functioning of complex systems. Discerning resilience concepts in psychology and psychiatry might contribute to a mechanistically appropriate characterization of the complexity inherent in bipolar disorder and mental illnesses at large. Such distinctions can also help to contextualize and unify clinical practice (preventative vs. reactive treatment) within the broader systemic functioning of mental illnesses.
